# Supplementary material for: Commentary: CRISPR-Cas systems against carbapenem resistance—from proof-of-concept to clinical translation
Source: Front Microbiol. 2026 Mar 2;17:1773181. doi: 10.3389/fmicb.2026.1773181 (PMC12990143; doi:10.3389/fmicb.2026.1773181)
Supplement: Supplementary file 1 [file Data_Sheet_1.pdf]

Commentary: CRISPR–Cas systems against carbapenem resistance — from proof-of-concept to clinical translation.

Tsolakidou highlights the need for CRISPR-Cas therapeutics to be made "clinical-ready," "audit ready," and "equitable" throughout all health care delivery systems, in order for them to be successfully adopted [1]. As another topping opportunity, particularly for readers at Frontiers whose coastline encompasses both clinical practice and policy making, we are eager to utilize and refine the concept of "sequence-guided therapeutics." The goal of this implementation framework is to provide a clear means of evaluating CRISPR-based intervention technologies for their potential role in addressing AMR. The focus will be to produce stewardship bundles for CRISPR anti-AMR interventions that maintain the same seriousness of evaluation as a traditional drug or biologic, using relevant metrics for the risks and benefits associated with these types of interventions. Through this translational lens, we see the CRISPR-based interventions transforming our understanding of how we evaluate the potential for resistance. Instead of relying solely on the traditional definition of bacterial resistance as the absence of an effective drug, CRISPR interventions also introduce new ways for bacteria to escape from the intended target of the intervention; either by making changes to the surface receptors (e.g. mutations) or through means of altering the genetic coding of the gene(s) targeted by the intervention (e.g., due to the loss of payloads, mobile Genetic elements).

This commentary provides an interpretive synthesis for translation; the structured "CRISPR–AMR stewardship bundle" is explicitly offered as a proposed minimum reporting heuristic (i.e., a practical checklist to improve comparability), not as a consensus guideline, clinical recommendation, or regulatory standard. It is intended to stimulate harmonization and implementation-ready reporting across settings.

Terminology (used consistently throughout): "sequence-guided therapeutics" refers to interventions whose target selection, triage, and monitoring are anchored to pathogen sequence features (genes/variants) rather than phenotype alone; "programmability" refers to the capacity to retarget or update the intervention by sequence design; and "escape" denotes loss of effect via genetic or ecological routes (e.g., target mutation, receptor change, payload loss), with uncertainty that necessitates surveillance and iterative adaptation.

Recent data show both progress in and challenges with the use of engineered bacteriophages as delivery vehicles containing antisense CRISPR-Cas systems to eliminate specific pathogenic bacteria in vivo[2]. This expands the potential for the use of these engineered phages for application in vivo, beyond just an in vitro test of feasibility. Furthermore, the ideas about being able to edit bacteria "in situ," i.e. directly, in their natural environment, appear as though they are a realistic possibility. However, advanced use of these technologies

will require additional work to ensure the appropriate specificity, therapy delivery, and unintended environmental consequences are all monitored closely [3].

The most interesting aspect of the carbapenem rescue initiative is that the use of bacteriophage capsid to deliver gene-specific inhibitor to the bacteria could lead to improved or "sparing" use of antibiotics instead of a direct bacteriolytic substitute [4]. The results presented here strengthen the overall conclusion of the previous review [1] while highlighting a continuing problem in the area of CRISPR technology: a lack of consistency in data reporting which creates difficulty in comparing different platforms for their effectiveness at preventing resistance and predicting clinical applicability. A clinical pathway for CRISPR-based stewardship of AMR requires four basic elements that can be packaged together into a CRISPR-AMR stewardship bundle.

To avoid overreach for this article type, we reiterate that the bundle below functions as a platform-agnostic but parameter-sensitive reporting heuristic: it can be applied across diverse CRISPR modalities (e.g., Cas9 vs Cas13, nuclease vs base editing, replicative vs non-replicative vectors, and different delivery vehicles), while allowing the reported parameters to be tailored to each modality and infection niche. The cited studies are used illustratively to motivate these reporting minima, not as a definitive evidence synthesis or endorsement of any single platform.

1. Indication triage + minimal diagnostics

CRISPR anti-AMR programs should predefine a minimal diagnostic set: which gene(s)/variant families qualify a case, what turnaround time is required, and what to do in mixed infections. Without explicit minimal diagnostics, CRISPR risks becoming a therapy only for high-resource sequencing centers—undermining global relevance.

2. Delivery accountability (dose-at-site logic)

Because efficacy depends on payload delivery to the infection niche, manuscripts should standardize reporting of: (i) delivered dose, (ii) site-of-infection exposure, (iii) persistence, and (iv) microbiome perturbation. Preclinical demonstrations are increasingly sophisticated [2], but translational comparability demands consistent "dose-at-site" reporting rather than platform-specific proxies.

3. Escape forecasting + response plan

CRISPR platforms must treat escape as predictable biology, not an afterthought. A minimal plan should include: expected escape mechanisms, monitoring windows, thresholds for action, and a programmable "second-line" response (e.g., multiplex targeting, spacer cycling, or combined adjuvant strategies). The delivery-yield constraints and spacer dependence observed in gene-reversal work are precisely the kind of variables that should be operationalized into monitoring standards [4].

We temper the framing here: escape mechanisms are often predictable in class (the "how"), but not reliably predictable in frequency, timing, or dominance in vivo (the "how much/when"), which are context-dependent. Accordingly, the bundle

emphasizes prospective surveillance, prespecified action thresholds, and an adaptive response plan rather than forecast accuracy or certainty.

4. Equity-by-design

The concept of programmability will not have any real-world value, on a worldwide basis, until manufacturing processes, cold chain delivery systems, and diagnostic testing methodologies can be brought out of the specialty facility realm into the general population. In conjunction with this, design choices made to minimize the potential ecological impact and maximize the potential for successful deployment of programming technologies (e.g., non-replicative development and appropriate controls placed on the behaviour of the genetic cargo), should be clearly communicated as a necessary component of the translation process, rather than just as optional ethical commentary [3,5]. The target panels that have been established should also represent regional carbapenemase epidemiology, rather than simply reflecting the priorities of one geographic location.

Equity-by-design is therefore treated as an early design constraint rather than an immediate equity outcome: the manuscript argues for pre-specifying access-relevant requirements (diagnostic turnaround, cold-chain tolerance, manufacturing complexity/cost, and fit for decentralised care) early enough to shape platform development, without claiming that any single design choice guarantees equitable deployment. Practical pathways include modular diagnostic packages (from rapid gene panels to sequencing where available), tiered manufacturing and distribution strategies (including temperature-stable formulations where feasible), and transparent reporting of logistics that determine deployability in low-resource settings.

Likewise, the call for region-specific carbapenemase target panels should be interpreted as modular customization within a shared global framework (swappable, locally validated modules), not bespoke redesign of the entire platform; this approach supports global interoperability while respecting regional epidemiology and supply-chain realities.

In Tsolakidou's overview, CRISPR-Cas has been identified as a potential solution for "post-antibiotic anxiety," and an innovative option for creating personalized medicines[1]. The next logical step in this area is to implement this new technology by aligning the goals of innovative science (Scientific Purpose) with the implementation (Clinical Application) of that science (i.e. How and Why we will use it). By establishing the minimum expectations for all of these four areas, including: a minimum standard of care for diagnostics, delivery & compliance, post-market surveillance, and equitable access to these treatments (Equity by Design) as one set of criteria (Stewardship Bundle), establishing those criteria as minimum standard expectations for research publications would allow for rapid translation of clinical research into clinical practice while also providing researchers with comparable results across multiple institutions globally

For clarity of translation, each bundle element answers a practical "why it matters" question: (i) indication triage protects clinical appropriateness and turnaround; (ii) dose-at-site logic protects reproducibility across delivery platforms; (iii) escape surveillance protects durability and safety; and (iv) equity-by-design protects deployability beyond high-resource centres. This signposting keeps the piece within the scope of a commentary while making the implementation logic explicit.

References

1. Tsolakidou PJ. CRISPR–Cas systems against carbapenem resistance: from proof-of-concept to clinical translation. Front Microbiol. 2025;. doi:10.3389/fmicb.2025.1725247.
2. Gencay YE, Zambolin S, Bräsen C, et al. Engineered phage with antibacterial CRISPR–Cas selectively reduce Escherichia coli burden in mice. Nat Biotechnol. 2024;. doi:10.1038/s41587-023-01759-y.
3. Brödel AK, Jaramillo A, Isalan M, et al. In situ targeted base editing of bacteria in the mouse gut. Nature. 2024; doi:10.1038/s41586-024-07681-w.
4. Kawaguchi T, et al. Gene-specific reversal of carbapenem-resistant Pseudomonas aeruginosa via phage-delivered CRISPR-Cas13a. npj Antimicrob Resist. 2025; doi:10.1038/s44259-025-00167-3.
5. Mayorga-Ramos A, et al. CRISPR-Cas-based antimicrobials: design, challenges, and therapeutic potential. ACS Infect Dis. 2023;. doi:10.1021/acsinfecdis.2c00649.
